# Plasticity in the Rat Prefrontal Cortex: Linking Gene Expression and an Operant Learning with a Computational Theory

**DOI:** 10.1371/journal.pone.0008656

**Published:** 2010-01-11

**Authors:** Maximiliano Rapanelli, Sergio Eduardo Lew, Luciana Romina Frick, Bonifacio Silvano Zanutto

**Affiliations:** 1 Instituto de Biología y Medicina Experimental (IBYME-CONICET), Laboratorio de Biología del Comportamiento, Ciudad de Buenos Aires, Buenos Aires, Argentina; 2 Instituto de Ingeniería Biomédica, Universidad de Buenos Aires, Ciudad de Buenos Aires, Buenos Aires, Argentina; The Mental Health Research Institute of Victoria, Australia

## Abstract

The plasticity in the medial Prefrontal Cortex (mPFC) of rodents or lateral prefrontal cortex in non human primates (lPFC), plays a key role neural circuits involved in learning and memory. Several genes, like brain-derived neurotrophic factor (BDNF), cAMP response element binding (CREB), Synapsin I, Calcium/calmodulin-dependent protein kinase II (CamKII), activity-regulated cytoskeleton-associated protein (Arc), c-jun and c-fos have been related to plasticity processes. We analysed differential expression of related plasticity genes and immediate early genes in the mPFC of rats during learning an operant conditioning task. Incompletely and completely trained animals were studied because of the distinct events predicted by our computational model at different learning stages. During learning an operant conditioning task, we measured changes in the mRNA levels by Real-Time RT-PCR during learning; expression of these markers associated to plasticity was incremented while learning and such increments began to decline when the task was learned. The plasticity changes in the lPFC during learning predicted by the model matched up with those of the representative gene BDNF. Herein, we showed for the first time that plasticity in the mPFC in rats during learning of an operant conditioning is higher while learning than when the task is learned, using an integrative approach of a computational model and gene expression.

## Introduction

Computational theories have been widely used in order to study the emergent properties of neural circuits [1]–[2]. In this sense, several models have been designed to describe the neuronal mechanisms underlying visual tasks, feeding behavior, reward prediction and operant conditioning, among others, integrating different brain areas [3]–[6]. In a previous work, we proposed a computational theory to simulate learning of several tasks [7]. Given that the lateral Prefrontal Cortex (lPFC) in primates or medial Prefrontal Cortex (mPFC) in rodents, is involved in cognitive processes such as goal-directed behavior, working memory, executive control and reward information [8]–[9]. The lPFC is a key element in complex behaviors, as for example, perceptual categorization and matching to sample. For this reason, we included the lPFC to improving the model for other tasks [7]. One of the predictions of this model is that neural plasticity activity is higher in the lPFC while animals are actually learning an operant conditioning task rather than after it has been learned. In our model, synaptic plasticity modifications are calculated as hebbian and anti-hebbian law, simulating long term potentiation (LTP) and long term depression (LTD), respectively. Therefore, this model is a behavioral and neurophysiological plausible neural network representation; however, it has not been yet confirmed by biological evidence. Knowledge of the molecular mechanisms underlying task learning would be useful to verify and fit plasticity computations in the model. An accepted approach to indirectly determine synaptic plasticity *in vivo* is to measure transcriptional fluctuations of genes whose expression is deeply associated to synaptic plasticity. Neural plasticity is required for circuit formation, depends on bi-directional communication between pre and post synaptic neurons, dendrite and axonal branching and remodelling, among others [10]. There are several genes associated with plasticity, among which the most important are brain derived neurotrophic factor (BDNF), cAMP Response Element Binding Protein (CREB), Synapsin I, Calcium/Calmodulin protein kinase II (CamKII), activity-regulated cytoskeleton-associated protein (Arc), c-fos and c-jun. BDNF is the main protein in the brain involved in the activity-dependent neuronal plasticity, synaptic transmission and growth of dendrites and axons [11]. Moreover, regulation of BDNF secretion is related to LTP and LTD [12]. The transcription factor CREB is another crucial mediator of these processes that acts by regulating transcription of effector genes, including BDNF [13]. Synapsin I, a major component of synaptic vesicles, is known to be up-regulated by LTP in the dentate gyrus [14], and its transcription, which can be regulated by BDNF, is also associated to different degrees of learning [15]–[16]. In addition, CamKII plays a key role in neurotransmission, gene expression and plasticity [17]. The transcripts of CamKII isoforms are tightly influenced by LTP in the rat cortex [18] and it was observed that gene transcription and availability are regulated by BDNF [19]. Besides, immediate early genes (IEGs), like c-fos, c-jun and Arc, have been proposed as markers of neuronal activation [20], which are also regulated by BDNF [13], [21], [22]. It has been found that Arc expression is involved in spatial learning, exploration learning and selective reactivation of networks [23]. The AP-1 subunits c-fos and c-jun are closely related to learning processes, plasticity and neuronal activation in rat cortex and hippocampus [24]–[28].

The aim of this work was to confirm predictions of our previous model using an animal model, studying behavioral parameters and molecular markers of plasticity. For this purpose, we analyzed differential expression of genes related to plasticity and IEGs in the mPFC of rats during learning of an operant conditioning task. Moreover, these results were used to fit the theoretical model, indicating how to compute the synaptic weights.

## Results

### Behavioral Data

**Figure 1:**
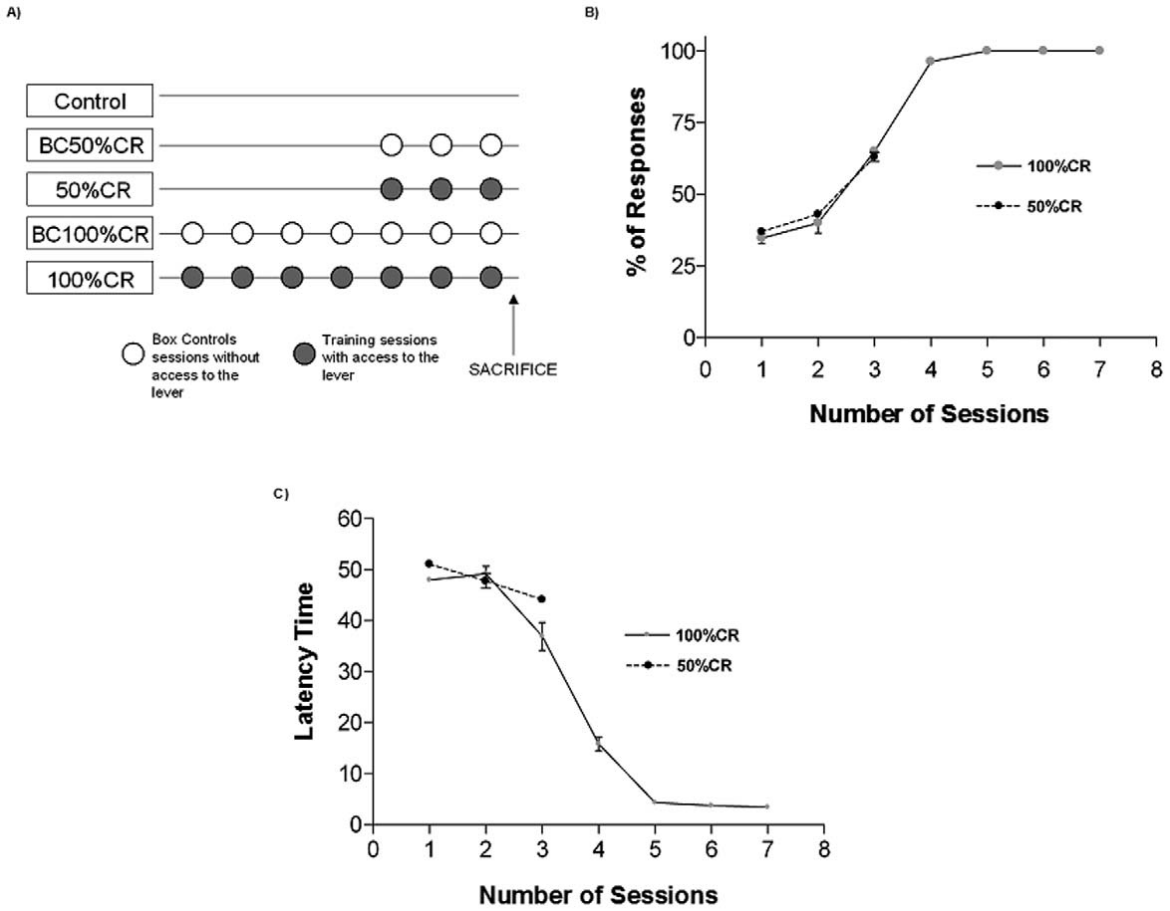
Scheme of behavioral procedures and sample extraction. Diagram of how trainings were performed and time of sample extraction (panel A). Animals from: Control, BC50%CR, 50%CR, BC100%CR and 100%CR groups, were synchronized to be sacrificed 15 minutes after the last session of 50%CR and 100%CR groups (panel A). Percentage of responses during training session is expressed as the mean ± S.E.M. number of responses in a training session of 25 trials (panel B). Latency time is expressed as the mean ± S.E.M. as the time that elapses between presentation of the conditioned stimulus and occurrence of the lever pressing (panel C). If no response was performed by the animal, it was the time until the end of the trial.

We used a skinner box to train animals within an appetitive operant conditioning, that is, where the animal presses a lever to receive a palatable pellet as reward. Behavioral parameters considered for measuring learning were latency response and number of correct responses. Animal groups were designed in accordance to the following criteria: 50–65% of correct responses (50%CR) and 100% of correct responses and latency time lower than 5 seconds for three consecutive sessions (100%CR). The trainings and sample extraction was performed as shown in Figure 1A. In the third session, animals from the 50%CR group reached 63% of correct responses (Figure 1B) and latency time of 44 seconds (Figure 1C), whereas animals from 100%CR reached 63.5% of responses (Figure 1B) with a latency of 40 seconds (Figure 1C). Moreover, animals from the 100%CR group reached 100% of correct responses and a latency time of 4 seconds in the fifth session. Consequently, animals belonging to the 100%CR group, performed 100% of responses with a latency time lower than 5 seconds in the 6^th^ and 7^th^ sessions (Figure 1B and 1C). Control groups were Box Control of 50%CR (BC50%CR), Box Control of 100%CR (BC100%CR) and *naïve* (Control).

### Plasticity Gene Expression Is Increased during Learning

Aiming at the expression of genes related with neural plasticity in this learning paradigm, we measured their mRNA levels using Real Time RT-PCR in the mPFC from animals that reached 50–65% of responses (50%CR) or 100% of responses and a latency time lower than 5 seconds (100%CR), as defined in Figure 1, and compared them to their respective controls (BC50%CR and BC100%CR). All mean differences were evaluated by ANOVA followed by post hoc Tukey's Multiple Comparisons Test for group comparison.

**Figure 2:**
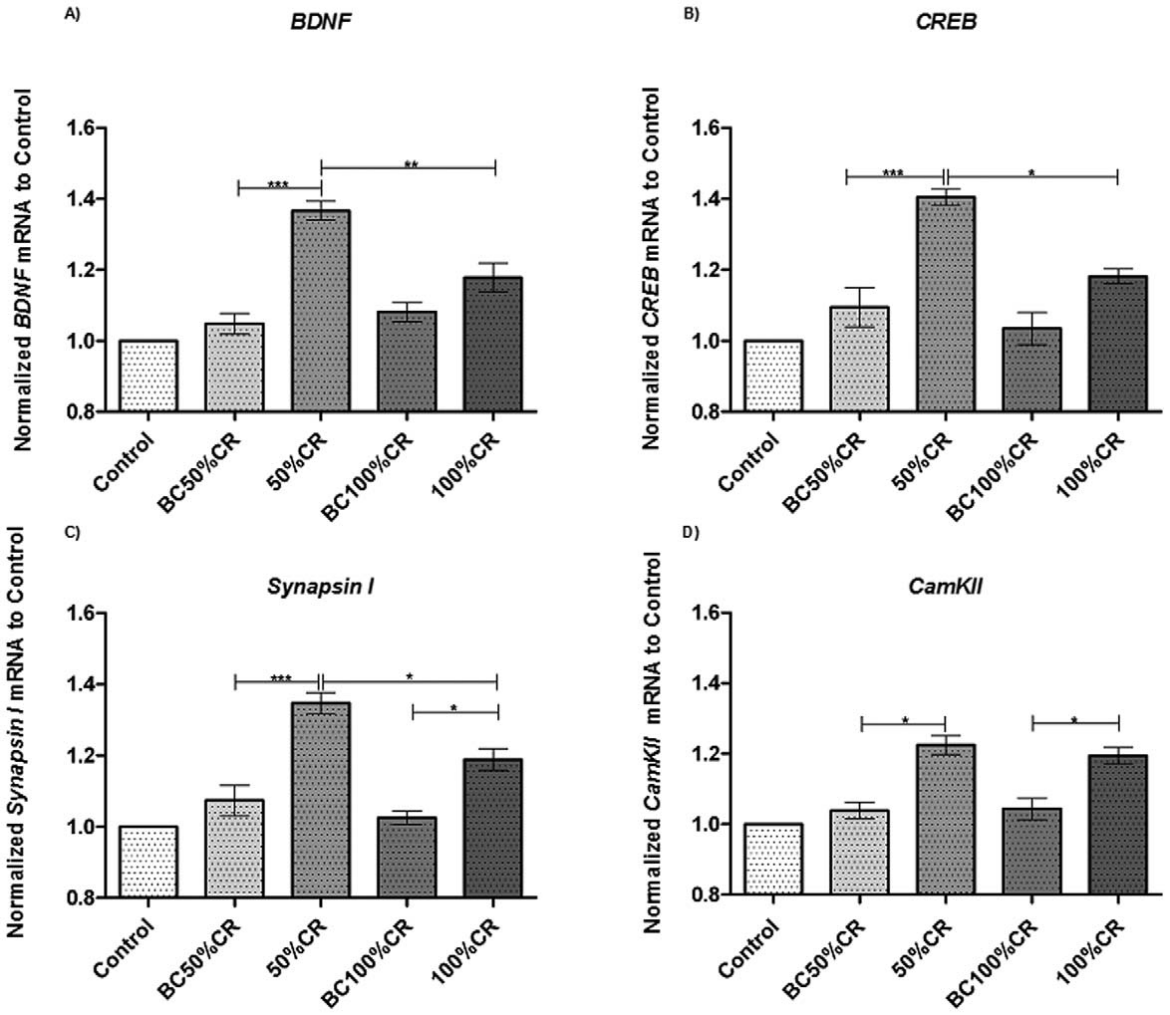
Modification of plasticity related genes levels. Differential expression of BDNF (panel A), CREB (panel B), Synapsin I (panel C) and CamKII (panel D) in the mPFC due to learning. Values are expressed as a percentage of the cage control value (100%), and represent the mean ± S.E.M. Control group (n = 6); BC50%CR, Box Control 50%CR (n = 6); 50%CR (n = 6); BC100%CR, Box Control 100%CR (n = 6), 100%CR (n = 6). *P<0.05, **P<0.01, ***P<0.001. One way ANOVA followed by Tukey's post hoc test.

The first plasticity related gene studied was BDNF, which showed a significant difference between means [*F*(4, 25, 27.25), p<0.0001]. Comparisons put into evidence that BDNF expression levels in the mPFC from 50%CR animals were increased by 31.9% (p<0.001) relative to BC50%CR (Figure 2A). However, no significant differences were found between 100%CR and BC100%CR animals, whereas for 100%CR animals, BDNF expression was decreased by 18.9% (p<0.01) when compared with 50%CR (Figure 2A).

On the other hand, a significant difference was observed for CREB [*F* (4, 25, 21.20), p<0.0001]. In this way, a comparison carried out later between 50%CR and BC50%CR groups resulted in a 31% increment (p<0.001) in 50%CR group (Figure 2B). Consequently, statistical analysis showed, that in 100%CR animals, CREB expression was significantly increased by 14.7% (p<0.05) with respect to the control group BC100%CR. CREB mRNA levels in 100%CR animals were reduced in 22.3% (p<0.05) with respect to 50%CR animals (Figure 2B).

In the case of Synapsin I mRNA levels in mPFC, statistical analysis resulted in a significant difference between means [*F* (4, 25, 25.55), p<0.0001]. Thereafter, an increment of 27.4% (p<0.001) was found between 50%CR and BC50%CR groups (Figure 2C). In addition, another increase of 16.3% (p<0.05) in Synapsin I expression was determined when a comparison between 100%CR and BC100%CR was performed (Figure 2C). Also, we found a decrease of mRNA levels of Synapsin I of 15.9% (p<0.05) when we contrasted 50%CR and 100%CR animals (Figure 2C).

Analysis of mRNA levels of CamKII in the mPFC led to a difference between mean groups [*F*(4, 25, 18.89), p<0.0001] and an increase of 18.7% (p<0.05) in 50%CR vs. BC50%CR groups (Figure 2D). Moreover, testing CamKII in 100%CR animals produced augmented mRNA levels by 15.2% (p<0.05) with respect to BC100%CR group (Figure 2D). Nevertheless, no significant differences were found in CamKII expression in the mPFC between 50%CR and 100%CR groups. It is worthy to note that in BDNF, CREB, Synapsin I and CamKII, no significant differences were found between control groups (Control, BC50%CR and BC100%CR).

### c-fos, c-jun and Arc Profile Expression in an Operant Conditioning Learning Task

**Figure 3:**
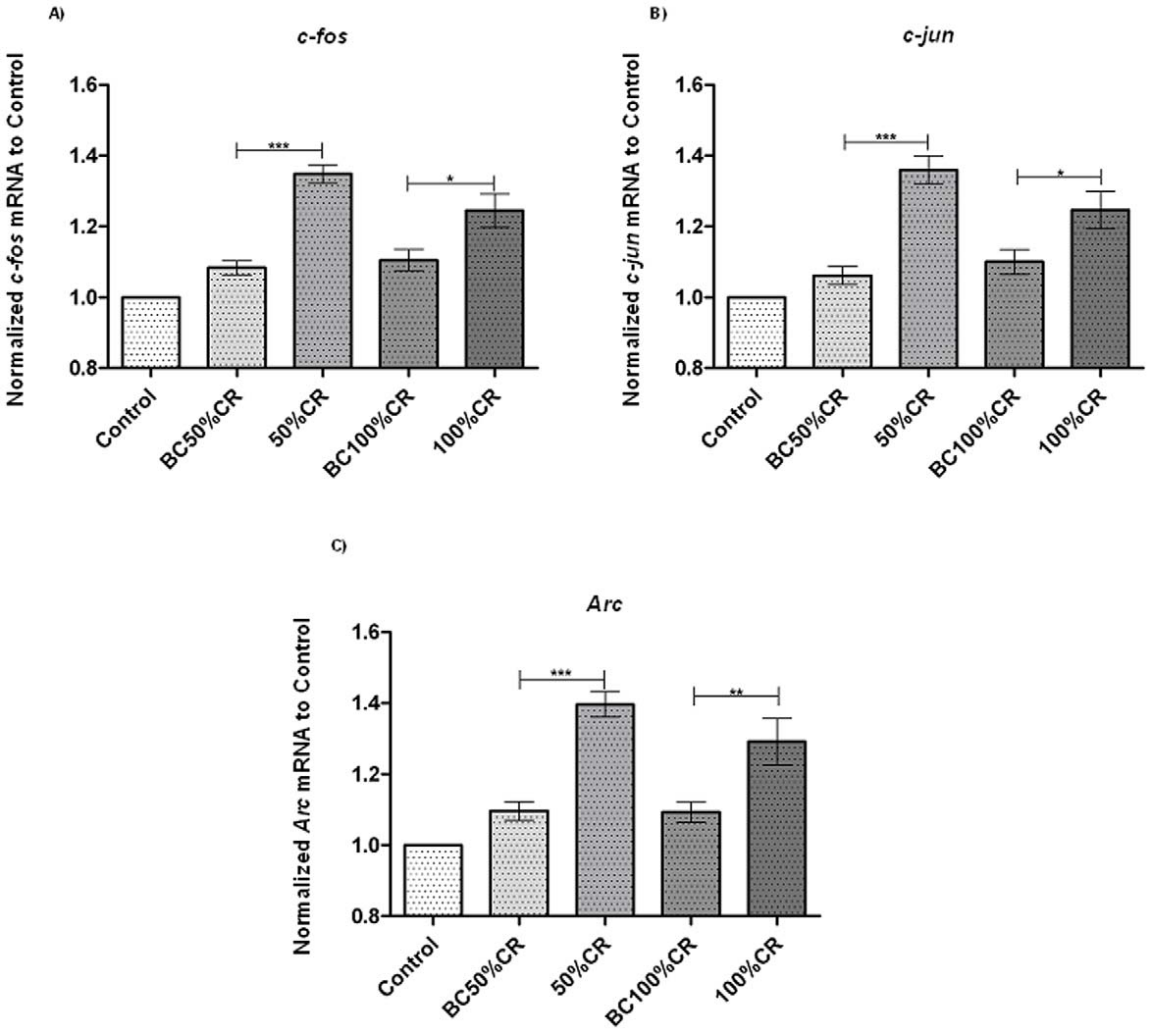
Changes in IEGs levels. Gene expression profile of c-fos (panel A), c-jun (panel B) and Arc (panel C) during learning in the mPFC. Experimental values are expressed as percentage of the cage control value (100%), and represent the mean ± S.E.M. C, Control group (n = 6); BCIT, Box Control IT (n = 6); IT (n = 6); BCTr, Box Control Tr (n = 6), Tr (n = 6). *P<0.05, **P<0.01, ***P<0.001. One way ANOVA followed by Tukey's post hoc test.

Another interesting group of genes to examine were the IEGs *c-fos*, *c-jun*, and *Arc*. First, we started analyzing the expression levels of *c-fos* by ANOVA and a significant difference between groups means was found [*F*(4, 25, 22.93), p<0.0001]. The foregoing results showed an increase of 26.5% (p<0.001) between 50%CR vs. BC50%CR animals (Figure 3A) and an increment of 14% (p<0.05) when pairing 100%CR with BC100%CR. However, the comparison between 50%CR and 100%CR resulted in no significant difference (Figure 3A).

The second gene studied was *c-jun*, for which a difference between means was found [*F* (4, 25, 17.35), p<0.0001]. The mRNA levels were increased by 29.8% (p<0.001) in the 50%CR as compared with BC50%CR (Figure 3B). Also, an increment of 14.2% (p<0.05) was found between 100%CR and BC100%CR (Figure 3B). However, the comparison of 100%CR vs 50%CR resulted in no significant difference (Figure 3B).

The last gene analyzed was *Arc* and, once again, we found significant differences between means of experimental groups [*F* (4, 25, 19.01), p<0.0001]. In the comparison between BC50%CR and 50%CR (Figure 3C), we found an increase of 30% (p<0.001). In addition, for 100%CR vs BC100%CR, an increment of *Arc* levels (p<0.01) was observed (Figure 3C). Finally, there was no significant difference regarding Arc mRNA expression when we compared 50%CR with 100%CR (Figure 3C). It is remarkable that in these three genes analyzed, no significant difference was observed between control groups, BC50%CR and BC100%CR.

Taken together, these results would indicate that plasticity in the mPFC is higher while animals are learning an operant conditioning task than once said task is completely learned, as evidenced by the differential expression of marker genes.

### Simulations

The above results confirm one of the predictions of our previous model: in the lPFC in primates or mPFC in rats, the synaptic plasticity is higher while learning than once an operant conditioning task is learned. The first version of the model did not comprise any biological evidence measured *in vivo*, indicating how to compute the synaptic plasticity in the lPFC. Instead, the synaptic changes in the lPFC were computed by a Hebbian and anti-Hebbian rule simulating LTP and LTD, respectively. Therefore, we used the results described above to compute the synaptic changes in the lPFC. The biological data for BDNF were compared with the model prediction in order to fit the synaptic changes to the plasticity processes proposed by changes in plasticity related gene expression. BDNF was chosen among the genes studied herein given that it is by far the most important molecule related to cognitive processes.

**Figure 4:**
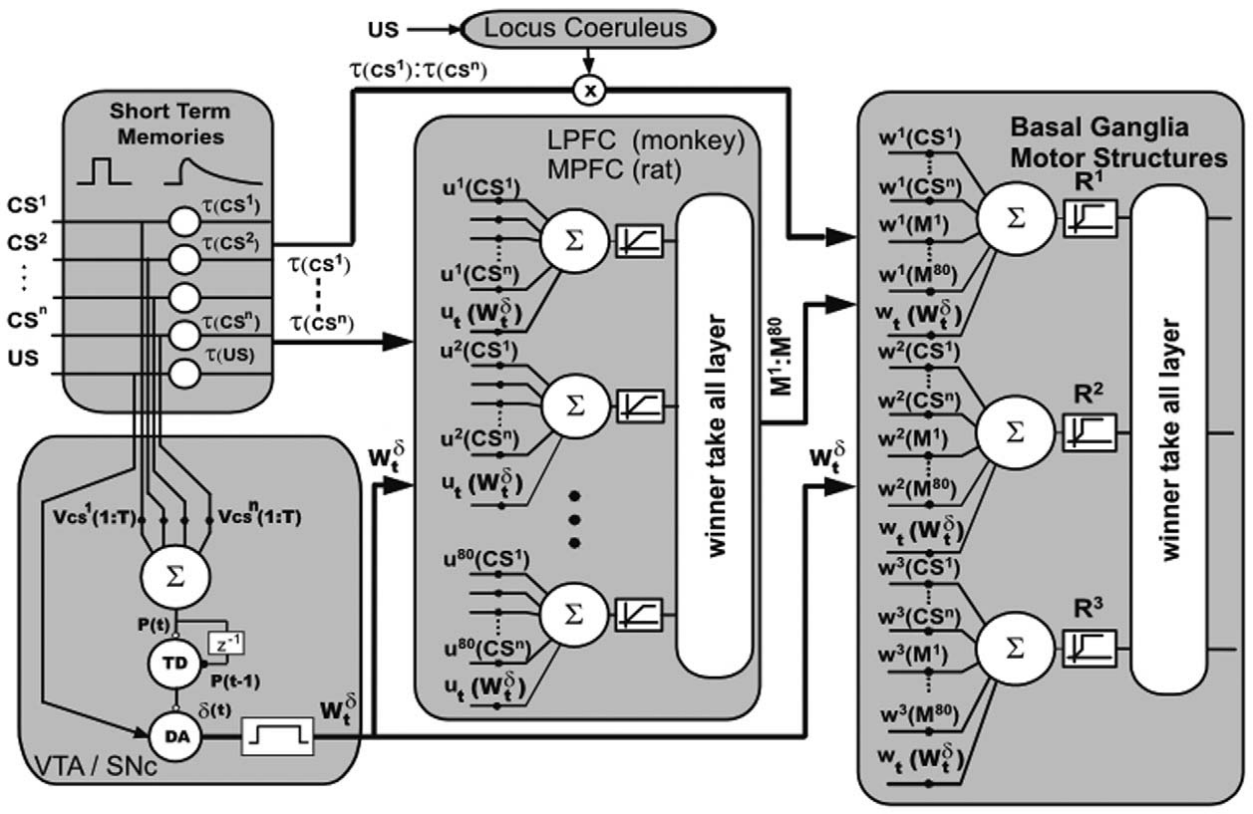
Scheme of the neural network model. The first layer generates short-term memories of the stimuli as a result of the interaction between different structures such as ventromedial PFC, inferotemporal ctx., posterior parietal ctx., hippocampus and amygdala. We used 80 neurons in the lPFC or mPFC for monkeys and rats respectively, 3 in the BG-PMC and a TD(lTD) model in the VTA/SNc. The Locus Coeruleus block represents a modulation exerted by the Locus Coeruleus over direct input-output synapses.

**Figure 5:**
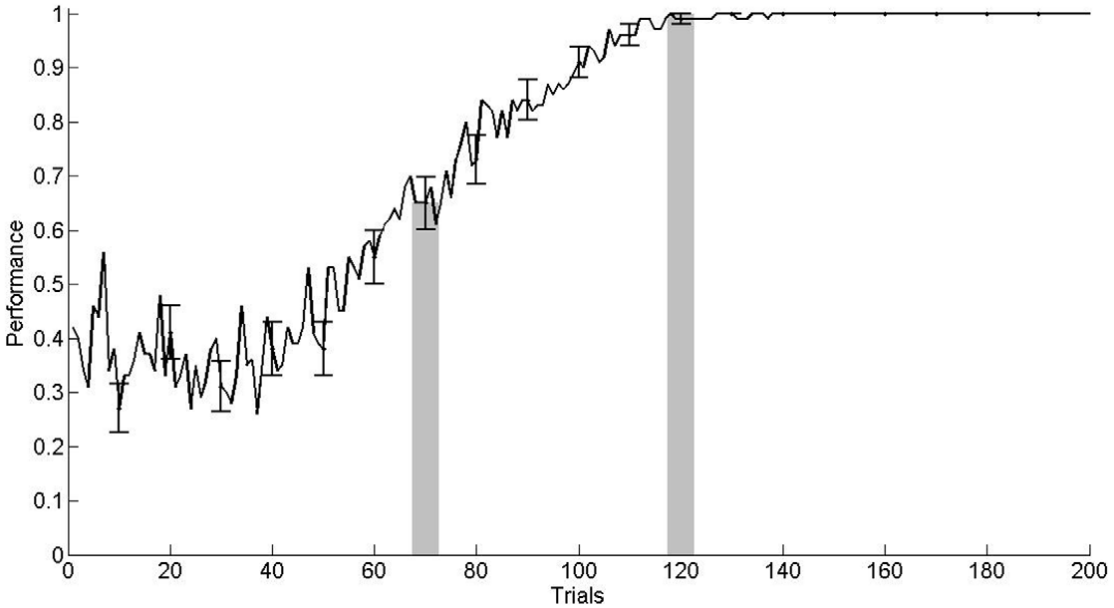
Averaged performance of the ensemble in operant conditioning learning. Error bars indicate the standard error to the mean performance and shadow bars shows the experimental performances obtained in Figure 1B.

**Figure 6:**
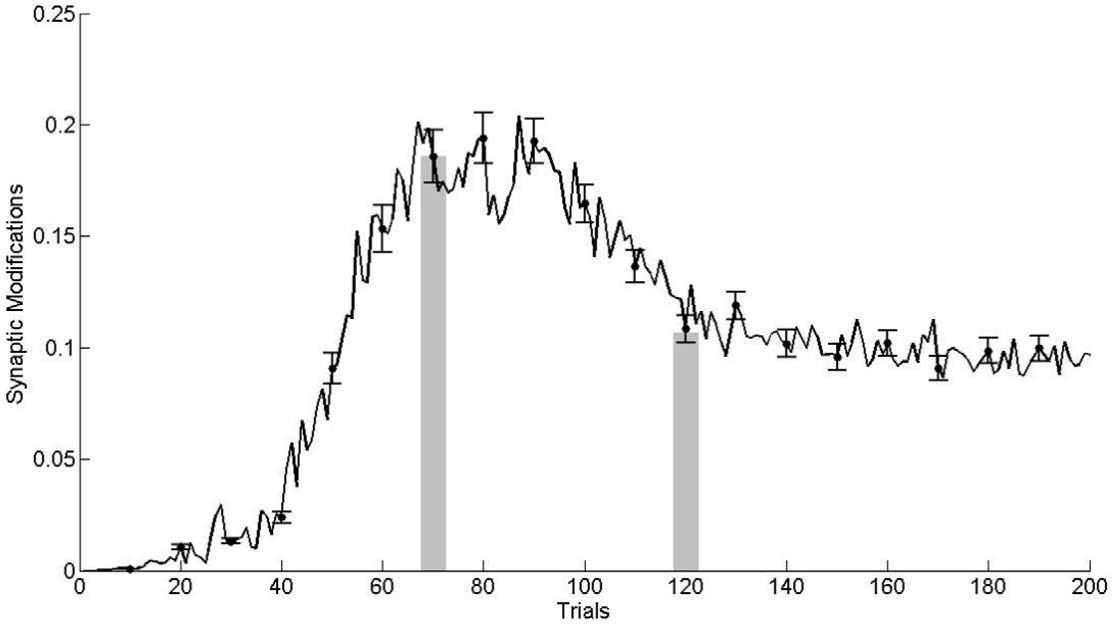
Averaged synaptic weights modifications during learning. Learning induces an increment of synaptic modifications that reach a maximum value near trial 80 and then the amount of change in synaptic weights decrease. At trial 120, the rate of change predicts the experimental result found in Figure 2A. Error bars indicate the standard error to the mean. Shadow bars shows the experimental values of BDNF obtained in Figure 2A.

In Figure 4 it can be observed a scheme of the neural networks model and the areas of the brain included. Statistical analyses were performed using an ensemble of 100 computational models. Each model was adjusted in the operant conditioning in the same way animals were trained. Model parameters were tuned to achieve 65% and 100% of performance in trials 70 and 120, respectively. Figure 5 shows the average performance for the model ensemble, wherein the performance obtained in the behavioral experiments of Figure 1 can be appreciated as shadow bars. During learning, neurons in the lPFC modified their synaptic weights according to the Hebbian or anti-Hebbian law (Figure 6). Modifications were expressed as the sum of the absolute value of LTP and LTD as a function of the training trials. Shadow bars indicate the expected plasticity increment and posterior decrement observed in Figures 2 and 3. As basal levels of BDNF were not included as a parameter in the computational model, the bar at trial 70 was fitted to the simulated average synaptic modifications, and the bar at trial 120 indicate the average value of BDNF obtained in Figure 2. As it can be observed, the dynamic of synaptic weight modifications predicted those values of BDNF found in the experimental results obtained when expression levels of marker genes in the rat mPFC were determined.

## Discussion

In a first approach run *in vivo*, we showed that mRNA gene expression related to plasticity is differentially modified during the course of learning of an operant conditioning task. At a first stage, all genes studied herein are up-regulated in the mPFC of animals that belong to 50%CR. Instead, in animals from 100%CR, these increments are lower.

We previously described a theoretical model [7] proposing a behavioral and neurophysiological plausible neural network that relies on a layer with short term memory (STM) traces, a reward prediction neural cluster, the PFC layer, and the layer with possible responses. In this model, the lPFC synaptic modifications were computed by Hebbian or anti-Hebbian law depending on the level of released dopamine, simulating LTP and LTD. Using this approach, we predicted that, in the lPFC, the synaptic plasticity is higher while learning an operant conditioning task than after it is learned.

Therefore, the biological evidences shown here support our previous model that proposed for the first time this prediction. This hypothesis is further supported by recent findings from our group, which demonstrated a differential gene expression in the hippocampus during learning of an operant conditioning task [29]. We observed higher hippocampal levels of plasticity markers while learning, followed by a decay of the plasticity markers when the task was learned. Taking together all these results, we propose that plasticity is higher while learning than when the task is already learned. Nevertheless, the plasticity predicted by the previous model qualitatively did not match the plasticity levels proposed by the experimental results.

A growing body of evidence has proposed BDNF as the leading neurotrophin that orchestrates learning and memory processes [30]. Previous articles showed that regulation of BDNF secretion is related to LTP and LTD and that BDNF regulates the different phases of LTP [12], [31], [19]. Moreover, BDNF increased transcription is the cause or the result of LTP induction [30]; therefore, it is a very important marker for plasticity.

The results presented here for BDNF mRNA levels in the mPFC suggests that BDNF plays an important role in the plasticity necessary to induce the modifications in neural circuitry.

However, BDNF is not the only responsible factor for plasticity in neuronal circuitry; it exerts control over several genes related to plasticity that help to promote it. For this reason, we decided to study other genes also linked to plasticity. Incremented levels of CREB and c-fos found during training are remarkable observations, not only because of the effects that both have *per se*, but also for the fluctuations in the transcription levels that qualitatively matched up with the BDNF. Moreover, because CREB promotes transcription of BDNF and c-fos [13] and BDNF feedbacks over CREB [32], these results reinforce the idea of higher plasticity while animals are learning and a role of BDNF regulating the process.

On the other hand, another two important genes related to plasticity, Synapsin I and CamKII, were incremented during learning. In the case of Synapsin I and CamKII, we propose that increments represent that plasticity is occurring in both learning groups, since both mRNAs are actively transcribed and regulated during LTP [14], [18]. Moreover, because it is known that both genes can be under the influence of BDNF [19], [15] and these levels coincided qualitatively with BDNF levels found in the present work, we propose that BDNF could be regulating both genes to allow plasticity.

A growing body of evidence indicates that Arc is a key player for long term depression (LTD) and LTP consolidation through BDNF signaling [21]. Here, we observed strong increments of Arc at different stages of learning that correlate with the incremented levels of BDNF. In this way, Arc could be intensively expressed by the influence that BDNF exerts over it. However, this result does not discard Arc as an important factor for plasticity, since that knock-out mice for Arc failed to consolidate synaptic plasticity and memories [33]. On the other hand, the increments of c-fos and c-jun in animals that learned the task and those that were learning the task, suggests a potential role for the transcription factor AP-1, as it was previously described for another paradigm [28]. Indeed, c-fos is under transcriptional control of CREB, which is under influence of BDNF; consequentially, BDNF is participating in c-fos enhanced transcription. Moreover, transient increments of c-jun mRNA were associated with early and late LTP [34]. Thus, the present results further confirmed that plasticity is higher while the animals are learning than when the animals learned the task. These increments in the different mRNAs suggest that there was major plasticity modifications while learning and begun to decline after learning; besides, cellular activation is higher during learning. Thus, we propose that in the beginning of learning there are major plasticity changes due to massive modifications of the pre-existent neural circuits and that when the task is learned, the remaining plasticity is more related with a late establishing and refinishing of neural circuits. In fact, functional magnetic resonance imaging studies in humans have shown that during brain processing of goal directed behaviors and other tasks that involves reward, the lPFC regions are more activated during learning and one of these studies went even further by showing that the lPFC has a hierarchical organization for controlling emotions and cognitive control in decision making [35], [36], [37].

The previous *in silico* model simulated a similar pattern of plasticity changes during learning, but did not matched up with the gene increment values measured in the mPFC. Therefore, we decided to change and to improve how the model computes the plasticity. Taking together previous findings described above [11] and the results presented here, we chose BDNF as the representative gene for plasticity to compared with the computed plasticity. It is important to remark that all genes measured here are reliable markers for measuring plasticity and that here we are not measuring synaptic plasticity *per se*. Instead, here we showed that plasticity processes are occurring differentially during learning.

In the previous version of the model, the plasticity in dopaminergic neurons was computed by the TD model. It reproduces dopamine neuron activity in many behavioral situations, but in that version we lacked experimental data supporting how to compute the synaptic plasticity in the lPFC of primates or mPFC in rats. Knowing that there is LTP and LTD, we computed the synapses plasticity by the Hebbian and anti-Hebbian, respectively. Based on previous data of BDNF gene expression, we have verified in the model how to compute the synaptic changes and fit the parameter of the synaptic weight of the lPFC. Herein, we showed that Hebbian and anti-Hebbian rules are suitable to simulate the synaptic plasticity in the lPFC. Interestingly, experimental results give us important information about these results to the model and to made even more reliable the plasticity predicted by the model. On the other hand, we showed the molecular mechanisms underlying learning an operant conditioning task in the mPFC and the control that exerts BDNF over other genes related to plasticity.

Finally, in a holistic integration, we have demonstrated that plasticity in the mPFC is higher during learning an operant conditioning task than once it is learned, by two completely different approaches: computational and experimental, obtaining concordant results. These results demonstrates for the first time that learning an operant conditioning task requires while learning massive modification of the neural circuits, whereas when the task is already learned, process decreases and is more related to late establishing of the neural circuitry.

## Materials and Methods

### Experimental Procedures

All experimental procedures were approved by the ethics committee of the *Instituto de Biología y Medicina Experimental-Consejo Nacional de Investigaciones Científicas y Técnicas* (IByME-CONICET) and were conducted according to the NIH *Guide for Care and Use of Laboratory Animals*.

### Animals

Two month old male *Long Evans* rats (300–325 g) were provided by the IBYME-CONICET, maintained on a 12 h light/dark cycle with food and tap water available *ad libitum*.

### Gene Expression during Operant Conditioning

#### Operant conditioning task

All behavioral procedures were performed during the light cycle and the operant conditioning task was performed in a standard operant chamber (MED Associates Inc, St. Albans, Vermont, USA) equipped with an input (DIG 710/711) and output (DIG 720/721/722) card for data acquisition and processing, one automated retractable lever, white light house, context red light, white noise and automated feeder. All animals included here were single housed and handled every day for at least 12 days. At the beginning of the experiments, rats were then food restricted for 3 days before training and throughout the experiment to maintain ∼80% of their *ad libitum* body weight. Three days of habituation followed. Rats were first placed in the training room for 15 min followed by a 20 min exposure in the operant chamber. During the habituation process, rats in the operant chamber were only exposed to context red light and white noise, and fed with 25 pellets (45 mg, BioServe) given randomly by the automated feeder. Two daily sessions of 25 trials were performed. To avoid changes in animal performance due to light cycle, the first session was performed between 8am–10am and the second between 3pm–5pm. A session started with the lever retracted, a house white light on and a red context light that remained on during all the session. Each trial begun when the lever came out for 60 seconds and white light turned off, if the animal pressed the lever received a pellet as a reward. The action of pressing the lever was considered as a response. When the trial ends, the white light turns on and the lever retracts for 20 seconds. If the animal did not push the lever during trial, no reward was given. Control animals remained in the bioterium during experimental procedures. BC50%CR and BC100%CR sessions started with the house white light on and a red context light on, thereafter, the white light turned off and the animal remained in the box with the lever retracted until 50%CR and 100%CR finished their training sessions. One experimental group criteria was to reach 50–65% of responses (50%CR) and the other one was to reach 100% of responses and a latency time below 5 seconds for three consecutive sessions (100%CR). Latency is calculated as the amount of time that elapses between presentation of the conditioned stimulus and occurrence of the lever pressing. If no response was performed, latency was the time until the end of trial (in our case 60 sec). Experimental groups were as follows: 50%CR (50%CR, n = 6), Box Control of 50%CR (BC50%CR, n = 6), 100%CR (100%CR, n = 6), Box Control of 100%CR (BC100%CR, n = 6) and Control (Control, n = 6).

#### Quantitative real-time reverse transcription polymerase chain reaction (RT-PCRs)

Fifteen minutes after completion of the last training session, all experimental and control rats were simultaneously killed by cervical dislocation and the brains were immediately removed. The mPFC was dissected and stored at −70°C. Frozen tissues were homogenized in Trizol Reagent (Invitrogen) and total RNA was purified. First strand complementary DNA (cDNA) was synthesized by retrotranscription using oligodT primers and SuperScript™II Reverse Transcriptase (Invitrogen). Real-time RT-PCRs were conducted in a GeneAmp 7500 Sequence Detection System (Applied Biosystems, Foster City, California, USA) and cDNA amounts per sample were determined using SYBR Green PCR Core Reagents kit (Applied Biosystems). All RT-PCR quantification procedure was performed in duplicates and was subjected to a heat dissociation protocol following the final cycle of the PCR to diminish unspecific products. Progression of PCR products and reaction were assessed by changes of the SYBR green dye fluorescence attached to double strand DNA. All values were normalized to *β–actin* as no significant differences were observed among groups of treatment when using other housekeeping genes [38].

Primer sequences (Invitrogen) were designed using Primer Express software (Applied Biosystems). Oligonucleotide sequences were: *Arc* forward: 5′-ACCGTCCCCTCCTCTCTTGA-3′; *Arc* reverse: 5′-GGCACCTCCTCTTTGTAATCCTATT-3′; *β-actin* forward: 5′-CAACTTGATGTATGAAGGCTTTGGT-3′; *β–actin* reverse: 5′-ACTTTTATTGGTCTCAAGTCAGTGTACAG-3′; *BDNF* forward: 5′-AAAACCATAAGGACGCGGACTT-3′; *BDNF* reverse: 5′-AAAGAGCAGAGGAGGCTCCAA-3′; *CamKII* forward: 5′-CATCCTGAACCCTCACA TCCA-3′; *CamKII* reverse: 5′- CCGCATCCAGGTACTGAGTGAT-3′; *c-fos* reverse: 5′-CGCAGCGATCTTCATCAAAC-3′; *c-fos* forward 5′-TCCACTGCCTGGGACAGAA-3′; *c-jun* forward: 5′-CGGCCCCGAAACTTCTG-3′; *c-jun* reverse: 5′-GTCGTTTCCATCTTTGCAGTCA-3′, *CREB* reverse: 5′-GGGAGGACGCCATAACAACTC-3′; *CREB* forward: 5′-GCCTCTGGTGATGTACAAA CATACC-3′; *Synapsin I*forward: 5′-GCAAGTGTTGTGGCACTGACTAAG-3′, *Synapsin I* reverse: 5′-CTTCTGGACACGCACATCGT-3′. All results in BC50%CR, 50%CR, BC100%CR and 100%CR for each gene are expressed as a normalized percentage of the control group.

### Statistics

All the statistical analysis was performed using GraphPad Prism 4.00 (GraphPad Software, San Diego California USA). Values were expressed as means ± SEM and compared using repeated measures ANOVA and post hoc comparisons with Tukey's Multiple Comparisons Test, differences among experimental conditions were considered statistically significant when P<0.05.

### Model

A brief explanation about the computational model is described below, a detailed version of it can be found in [7].

Briefly, the activity of each neuron in the model represents the activity of a certain functional cluster of neurons. The time is discretized in steps representing 100 ms each. The input layer is constituted by a set of cue selective neurons that compute short term memories (STM) of input stimuli. Each time that 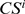 or *US_t_* are present, they are set to one, otherwise zero.

The output layer of the model contains 3 units, each of them is responsible of the execution of a behavioral response (R1, R2 and R3). If throughout a trial the activity of these output neurons does not exceed the activation threshold, a random response is executed with probability 1/3.

When a response is executed, the activity of its associated neuron is set to 1 along a period of 5 time steps, while the others are forced to 0 along the same time period.

In the simple task presented here, these responses represent pressing a key (R1) or doing any other response non-related with the task (R2, R3). All of them are codified at the motor-related structures layer.

Dopamine neurons have been shown to respond to unpredicted rewards [2]. Moreover, after repeated paired presentation of CS-US, neurons in midbrain dopaminergic structures as the Ventro Tegmental Area (VTA) and the Sustantia Nigra Pars Compacta (SNc), change their firing pattern codifying the prediction error of being rewarded. Time difference models (TD) [39] predict the firing of dopamine neurons for different paradigms (classical and operant) employing one or multiple conditioned stimuli.

In Figure 4, the VTA/SNc block is a TD model whose inputs are the CS's and the US.

A prediction of reward is calculated out from the set of stimuli present in each trial and the association between conditioned stimuli and reward is learned and coded in synaptic weights 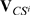 [1], [7].

Based on the reward and its predictions for each *CS^i^*, the prediction error *δ(t)* at time step *t* is computed. This prediction error is then used to update the synaptic weight vector 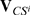 and to initiate a gating window 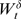 for learning mechanisms in PFC and BG-PMC.

When DA bursts occur, if *δ(t)>.θ_hebb_*, 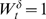 for the following T steps. The duration of this window (T) depends on the amplitude of *δ(t)* [7], in accordance with the experimental results obtained in [40]–[41].

When the predicted reward is omitted, DA firing goes below baseline. If *δ(t)<.θ_ant̃ihebb_*, 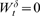 for the following 15 time steps. When the DA firing is close to baseline, i.e. *.θ_ant̃ihebb_<δ(t)<.θ_hebb_*, 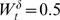.

Dopamine effects on neuron excitability had been widely shown. Dopamine decreases the spontaneous firing of PFC pyramidal neurons, mainly by exciting fast spiking inhibitory interneurons [42]. In the model, this inhibition is represented by clamped negative synaptic weight 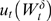 from the VTA to the PFC. On the other hand, the synergism between NMDA and D1 receptors could differentially change pyramidal neuron excitability based on the amount of extracellular dopamine [43]. In this sense, initially inhibited PFC pyramidal neurons will fire strongly when afferent inputs release large amounts of glutamate.

Neurons in the PFC respond according to the following,
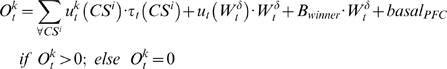
(1)where 
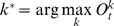
 represents the index of the winner neuron, *B_winner_* stands for the synergism between D1 dopamine receptors and NMDA receptors, and *basal_PFC_* is the baseline firing rate of PFC neurons.

A winner-takes-all mechanisms is simulated as follow
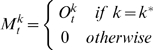
(2)


As in the PFC, the released DA inhibits the motor area through clamped negative synaptic weight 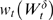 and, in contrast to this general inhibition, the winner neuron is excited proportionally to the released DA [44]. In this way, a “brake” is applied over all possible motor programmes and this motor program that surpasses a fixed threshold is released. The output of the response neurons is computed as,
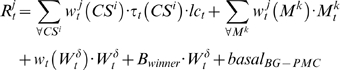
(3)where *basal_PFC_* is the baseline firing rate of BG-PMC neurons and 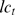 represents a modulation exerted by noradrenergic neurons of the Locus Coeruleus (LC) over visual and somatosensory cortical neurons [45], [7]. We model the tonic firing of LC neurons as a function of the received reward in a time window that includes many trials.
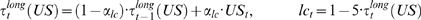
(4)


Short term memories for the response neurons are computed according to
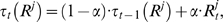
(5)and as in (8) for the PFC area, a winner-take-all rule is applied.

Dopamine effects on PFC pyramidal neurons are also related to modifications of synaptic efficacy via LTP and LTD. Previous models have used the DA signal in the modulation of synaptic weights modifications [46], [47], [7]. In our model, when 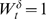, Hebbian learning is applied to both PFC and BG-PMC neurons. The opposite occurs when 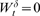. 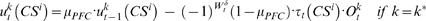
(6)

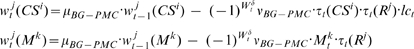
(7)where *μ_PFC_* and *μ_BG-PMC_* are first order momentum constants while *ν_PFC_* and *ν_GB-PMC_* are learning rates for the PFC and BG-PMC, respectively.
